# Clinical, radiologic, and serologic predictors of rheumatic disease in interstitial lung disease patients

**DOI:** 10.1007/s10067-025-07505-w

**Published:** 2025-06-11

**Authors:** Tugce Bozkurt, Elif Dincses-Nas, Sevilay Batibay, Zeynep Nilufer Tekin, Esen Kasapoglu

**Affiliations:** 1 Department of Internal Medicine, Division of Rheumatology, Istanbul Medeniyet University Goztepe Prof. Dr. Suleyman Yalcin City Hospital, Istanbul, Turkey; 2 Department of Radiology, Istanbul Medeniyet University Goztepe Prof. Dr. Suleyman Yalcin City Hospital, Istanbul, Turkey

**Keywords:** Connective tissue diseases, Idiopathic interstitial pneumonias, Interstitial lung disease, Rheumatic diseases

## Abstract

**Objective:**

Interstitial lung disease (ILD) can be the first manifestation of underlying rheumatic diseases. Identifying autoimmune features in ILD patients is crucial for early diagnosis and management. This study aims to evaluate the prevalence of rheumatic diseases in patients initially referred for ILD and to analyze their clinical, radiological, and serological characteristics.

**Methods:**

A total of 181 patients referred to the rheumatology outpatient clinic with suspected ILD, who had no known history of a rheumatologic disease, were retrospectively analyzed. Patients without chest CT/HRCT scans (n = 38) and those without a confirmed ILD diagnosis after radiological re-evaluation (n = 44) were excluded. Demographics, clinical symptoms, serology, and imaging findings were compared between groups.

**Results:**

Among the 99 ILD patients, 22 (22.2%) were diagnosed with a rheumatic disease following their ILD diagnosis. The most common rheumatic conditions were primary Sjögren’s syndrome (n = 7), systemic sclerosis (n = 5), and rheumatoid arthritis (n = 5). The rheumatic disease-related ILD (RD-ILD) group had a significantly higher female predominance (77.3% vs. 34.7%, p < 0.001) and lower smoking prevalence (p = 0.006) compared to the non-RD-ILD group. Usual interstitial pneumonia was the most frequently observed chest CT/HRCT pattern in both groups. ANA, RF, and ACPA positivity was significantly higher in RD-ILD patients (p = 0.029, p = 0.003, and p = 0.001, respectively). Two patients met the IPAF classification criteria, both exhibiting NSIP patterns on chest CT/HRCT.

**Conclusion:**

A substantial proportion of ILD patients were subsequently diagnosed with a rheumatic disease, highlighting the importance of routine autoimmune screening in ILD patients. Female predominance, lower smoking rates, and higher serological positivity in RD-ILD patients suggest that early rheumatologic evaluation could facilitate timely diagnosis and management.

****Key Points**:**

• *Interstitial lung disease may be the initial clinical sign of connective tissue diseases, highlighting the essential role of rheumatology in diagnosis and disease management*.

## Introduction

Interstitial lung disease (ILD) is associated with significant morbidity and mortality among patients with systemic rheumatic diseases including rheumatoid arthritis (RA), systemic sclerosis (SSc), primary Sjögren’s syndrome (pSS), idiopathic inflammatory myositis (IIM), and systemic lupus erythematosus (SLE), and may even be the first clinical manifestation of the disease [1]. Pulmonary function tests, physical examination and high-resolution computed tomography (HRCT) are commonly used and sufficient for diagnosis, however, in some of the cases, pathological confirmation may be required [2]. Histopathological patterns in connective tissue diseases (CTD)-related ILD include nonspecific interstitial pneumonia (NSIP), usual interstitial pneumonia (UIP), organizing pneumonia, desquamative interstitial pneumonia (DIP), lymphocytic interstitial pneumonia (LIP), and acute interstitial pneumonia [3, 4]. The primary pathological processes of ILD involve inflammation, fibrosis, or a combination of both [5].

Approximately 30% of ILD cases are related to CTD, and in some cases, ILD may be the first and only early sign of an underlying a rheumatic disease [6, 7]. In 2015, the term interstitial pneumonia with autoimmune features (IPAF) was defined for patients who have ILD and clinical, serological, or radiological signs of CTD but do not meet the classification criteria for CTD [8]. IPAF patients have a better prognosis than idiopathic pulmonary fibrosis patients; however, early treatment remains important to prevent permanent damage and even further to improve outcomes [9, 10]. Identifying an underlying rheumatic disease in patients presenting with ILD can be challenging, as clinical manifestations may be subtle or non-specific. Therefore, rheumatology consultation is crucial for the final diagnosis and management of these patients. This study aims to determine the prevalence of rheumatic diseases in patients initially diagnosed with ILD and to analyze their clinical, serological, and radiological characteristics.

## Materials and methods

### Study population

This study is a single-center, retrospective, cross-sectional, comparative observational study conducted at the rheumatology outpatient clinic of Istanbul Medeniyet University Goztepe Prof. Dr. Suleyman Yalcin City Hospital. A total of 181 patients with no prior diagnosis of a rheumatic disease were retrospectively evaluated at the rheumatology outpatient clinic between 2012 and 2023, after being referred by a pulmonologist with suspected ILD. Thirty-eight patients were excluded from the study due to unavailability of chest computed tomography (CT)/HRCT scans.

**Fig. 1:**
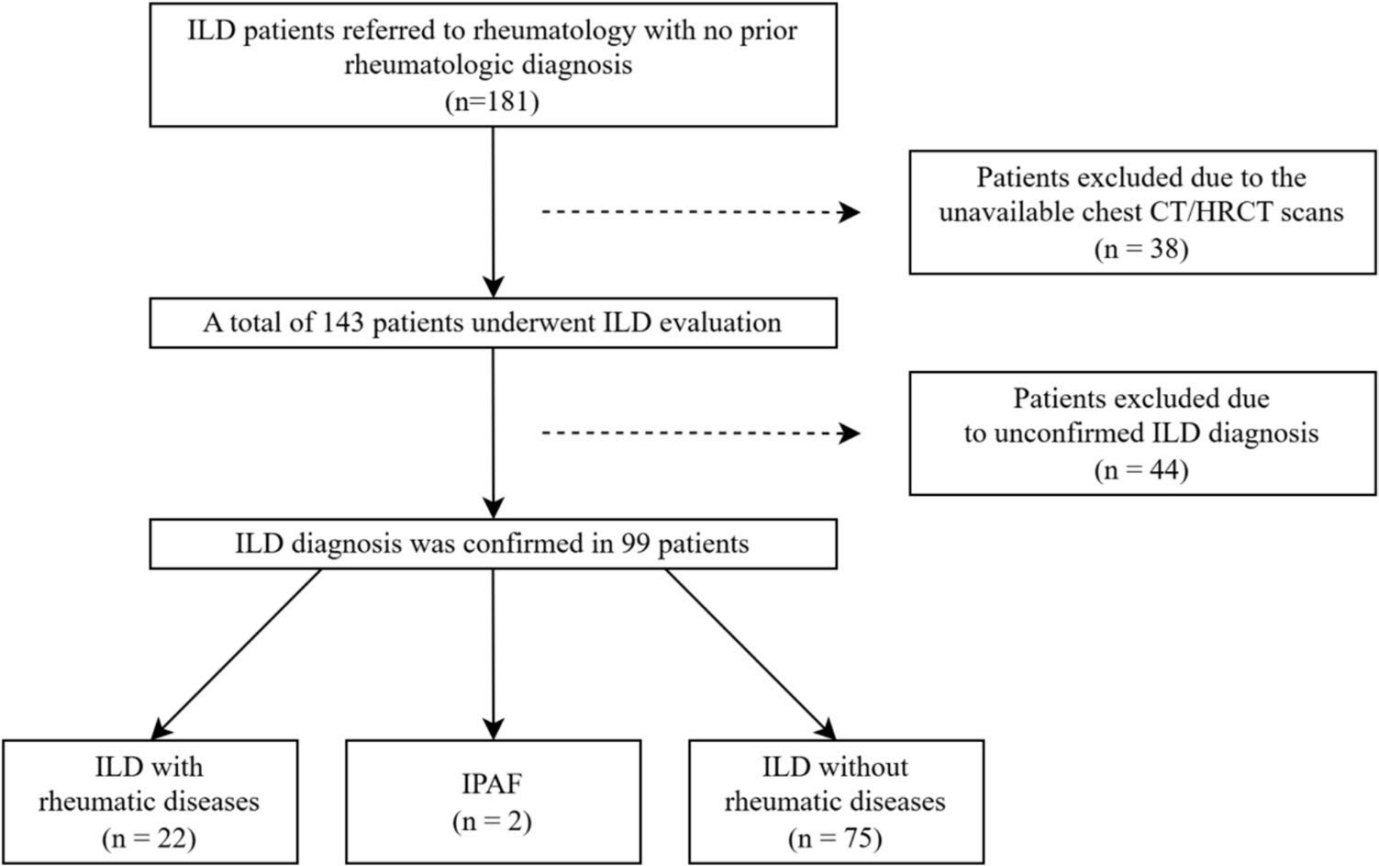
Evaluation and follow-up of patients with interstitial lung diseases. CT: Computed tomography, ILD: Interstitial lung disease, IPAF: Interstitial pneumonia with autoimmune features

The chest CT/HRCT images of the remaining 143 patients were evaluated by an experienced thoracic radiologist (ZNT), as shown in Fig. 1. Patients whose chest CT/HRCT patterns were classified as idiopathic interstitial pneumonia according to the American Thoracic Society/European Respiratory Society classification were analyzed for the presence of underlying rheumatologic diseases [11].

The diagnosis of rheumatic diseases in this study was established using internationally accepted classification criteria. Specifically, the 2010 ACR/EULAR classification criteria [12] were used for RA, the 2016 ACR/EULAR criteria for pSS [13], the 2013 ACR/EULAR criteria for SSc [14], the 2019 EULAR/ACR classification criteria for SLE [15], and the 2022 ACR/EULAR classification criteria for microscopic polyangiitis (MPA) [16]. For undifferentiated connective tissue disease (UCTD), the diagnosis was based on clinical judgment in the presence of autoimmune features and serologic positivity that did not meet the full criteria for a defined CTD. All rheumatologic evaluations were performed by experienced rheumatologists as part of routine clinical care. Although classification criteria were primarily used for the diagnosis of rheumatic diseases in this study, two patients (one with pSS and one with SSc) did not fully meet the classification criteria. However, both exhibited persistent clinical features and serological profiles strongly indicative of the corresponding diseases.

### Data collection

Demographic data, chest CT/HRCT images and reports, laboratory investigations (antinuclear antibody (ANA), extractable nuclear antigen (ENA) antibodies, rheumatoid factor (RF), anti-citrullinated protein antibody (ACPA), anti-neutrophil cytoplasmic antibody (ANCA), erythrocyte sedimentation rate (ESR), and C-reactive protein (CRP)), physical examination findings, pulmonary function test results, Schirmer test results, and minor salivary gland biopsy results were obtained from the patients’ medical records. ANA positivity was assessed by indirect immunofluorescence assay (IFA), with titers ≥ 1:100 considered positive. RF, ACPA, MPO-ANCA, and PR3-ANCA were measured using ELISA, while ENA antibodies were analyzed by immunoblotting. Results were interpreted according to laboratory reference cutoffs: RF > 12 IU/mL, ACPA > 17 U/mL, MPO-ANCA > 2 U/mL, and PR3-ANCA > 2 U/mL were considered positive. RF and ACPA titers were categorized as high when ≥ 3 times the upper limit of normal, and as low when below this threshold.

### Statistical analysis

Data analysis was performed using IBM SPSS Statistics, version 24.0. Categorical variables are presented as frequency (n) and percentage (%). According to data distribution, normally distributed numerical variables were presented as mean ± standard deviation (SD), while non-normally distributed numerical variables were presented as median and interquartile range, with the minimum and maximum values given in parentheses. Continuous variables were compared using Student’s t-test or Mann–Whitney U test, depending on data normality. Chi-square test and Fisher's exact test were used for categorical data, as appropriate. P-values less than 0.05 were considered statistically significant.

## Results

In the study, 76 (53.1%) of the 143 patients were female. The mean age of patients referred to the rheumatology clinic with a diagnosis of ILD was 64.9 (SD: 9.9) years. Cough was the most common symptom, reported by 57 patients (39.9%), followed by dyspnea in 39 patients (27.3%), and hemoptysis in just 2 patients (1.4%). Notably, 36 patients (25.5%) were asymptomatic. Sixty-eight patients (47.6%) had a history of tobacco use, while 37 patients (25.9%) had never smoked. Smoking history data were unavailable for the remaining 38 patients.

Chest CT/HRCT images of 143 patients were evaluated by the radiologist for ILD (ZNT). Chest CT/HRCT images of 99 patients (69.2%) demonstrated idiopathic ILD patterns according to the classification of Idiopathic Interstitial Pneumonias (IIPs) in the official statement of the American Thoracic Society/European Respiratory Society [11]. In contrast, ILD diagnosis of 44 patients (30.8%) was not confirmed. Among the 44 patients, chest CT/HRCT findings revealed various patterns, including hypersensitivity pneumonitis (n = 11), nonspecific findings that did not conform to the IIPs morphology (n = 9), infectious pulmonary disease findings (n = 8), pulmonary congestion (n = 4), combined pulmonary fibrosis and emphysema (n = 4), acute exacerbations of fibrosis (n = 3), emphysema (n = 2), cystic bronchiectasis (n = 2), and Langerhans cell histiocytosis (n = 1).

### Clinical characteristics of patients with ILD

A total of 99 patients were diagnosed with ILD, of whom 55 (55.6%) were male. The mean age at ILD diagnosis was 66.8 years (SD: 9.1).

While 31 (31.3%) patients were asymptomatic, cough was the most common symptom, affecting 40 (40%) patients, followed by dyspnea (n = 27, 27.3%). Additionally, 1 patient (1%) presented with hemoptysis. Among the 54 patients with a history of tobacco use (54.5%), no significant association was observed between smoking status and the presence of symptoms (p = 0.904).

The most common chest CT/HRCT pattern identified was UIP, observed in 70 patients (70.7%), including 54 with definite UIP, 11 with probable UIP, and 5 with indeterminate UIP. Additionally, 14 patients (14.1%) had NSIP, 11 (11.1%) had probable UIP or fibrotic NSIP, 2 (2%) had DIP, 1 (1%) had LIP, and 1 (1%) had respiratory bronchiolitis–associated ILD (RB-ILD). Both the patient with RB-ILD and the patient with DIP had a 50 pack-year smoking history.

Rheumatoid factor and ACPA levels were assessed in 79 and 73 patients, respectively. RF test positivity was detected in 8 patients (10.1%), while ACPA test positivity was observed in 5 patients (6.8%). ANA testing was positive in 36 (36.4%) patients, with the most frequent pattern being cytoplasmic (n = 9), followed by speckled (n = 6), nucleolar (n = 7), homogeneous (n = 4), granular (n = 5), and centromere (n = 2). Among 90 patients tested for ENA antibodies, 16 (17.8%) had positive results. The most frequently detected antibodies were anti-SSA (n = 4), anti-Scl-70 (n = 4), anti-Sm (n = 2), anti-dsDNA (n = 2), anti-SSB (n = 1), CENP-B (n = 1), anti-Ro-52 (n = 1), anti-Ku (n = 1), and anti-PmScl-100 and anti-Mi2 (n = 1). Additionally, among the 93 patients tested for ANCA, 4 (4.3%) were positive for MPO-ANCA, and 2 (2.2%) for PR3-ANCA.

A total of 22 (22.2%) patients were diagnosed with a rheumatic disease by a rheumatologist following their ILD diagnosis. Primary Sjögren’s syndrome (n = 7) was the most frequently diagnosed condition, followed by SSc (n = 5), RA (n = 5), SLE (n = 2), UCTD (n = 2), MPA (n = 1) (Table 1).

**Table 1:** Frequency of rheumatic diseases among patients diagnosed with ILD

ILD with rheumatic disease	n (%)
*Primary Sjögren’s syndrome*	7 (7.1)
*Systemic sclerosis*	5 (5.05)
*Rheumatoid arthritis*	5 (5.05)
*Systemic lupus erythematosus*	2 (2)
*Undifferrentiated CTD*	2 (2)
*Microscopic polyangiitis*	1 (1)
TOTAL	22 (22.2)

ILD: Interstital lung disease, CTD: Connective tissue disease

Two patients who met the IPAF classification criteria [8] were excluded from the analysis, and remaining patients’ clinical and demographic characteristics in the RD-ILD (n = 22) and non-RD-ILD (n = 75) groups were compared. Female predominance was significantly higher in RD-ILD patients (p < 0.0001). The mean age of the two groups was similar (64.5 vs. 67.6 years, p = 0.669). Smoking was more prevalent in the non-RD-ILD group (p = 0.006) (Table 2).

**Table 2:** Comparison of clinical, radiological, and laboratory findings between RD-ILD and non-RD-ILD patients

Patient characteristics and clinical findings	RD-ILD, n (%) n = 22	Non-RD-ILD, n (%) n = 75	p	All, n (%) n = 97
Female	17 (77.3)	26 (34.7)	< 0.001	43 (44.3)
Age at diagnosis of ILD (years, mean ± SD)	64.59 ± 9.3	67.6 ± 8.9	0.669	66.9 ± 9
Tobacco use (*n* = 74)*	8 (44.4)	44 (78.6)	0.006	52 (70.3)
* Current smoker*	5	27		32
* Former smoker*	3	17		20
* Never smoke*	14	8		22
* Unknown*	-	23		23
Chest CT/HRCT scans				
UIP	12 (54.5)	58 (77.3)	0.056	70 (72.1)
* Indeterminate UIP*	-	5 (6.7)		5 (5.1)
* Probable UIP*	2 (9)	9 (12)		11 (11.3)
NSIP	5 (22.7)	7 (9.3)	0.136	12 (12.4)
LIP	1 (4.5)	-		1 (1)
OP	-	-		-
Probable UIP or fibrotic NSIP	4 (18.1)	7 (9.3)		11 (83.5)
Desquamative IP	-	2 (2.7)		2 (2.1)
RB-ILD	-	1 (1.3)		1 (1)
Pulmonary function tests				
* DLCO % (n* = *45, mean* ± SD)*	61.2 ± 18.9	67.6 ± 19.7	0.921	65.6 ± 19.4
* FVC % (n* = 52, median [IQR])*	74.5 (50)	81 (32)	0.572	78.5 (36)
Pulmonary symptom	12 (54.5)	54 (72)	0.123	66 (68)
* Cough*	6 (27.2)	32 (42.7)		38 (39.2)
* Dyspnea*	6 (27.2)	21 (28)		27 (27.8)
* Hemoptysis*	-	1 (1.3)		1 (1)
CTD signs and symptoms				
* Xeroftalmia*	6 (27.3)	7 (9.3)	0.068	13 (13.4)
* Xerostomia*	8 (36.4)	8 (10.7)	0.008	16 (16.5)
* Raynaud phenomenon*	3 (13.6)	-	0.010	3 (3.1)
Laboratory findings				
ANA positivity	12 (54.5)	22 (29.3)	0.029	34 (35.1)
Rheumatoid factor (*n* = 79)*	6 (28.6)	2 (3.4)	0.003	8 (10.1)
ACPA (*n* = 73)*	5 (25)	-	0.001	5 (6.8)
MPO ANCA (*n* = 91)*	2 (10)	2 (2.8)	0.209	4 (4.4)
PR3 ANCA (*n* = 91)*	-	2 (2.8)	1	2 (2.2)
ESR (*n* = 88, median [IQR])*	40 (38)	23 (30)	0.177	24.5 (35)
CRP (*n* = 88, median [IQR])*	5 (11.5)	3 (3.9)	0.109	3 (4.8)
Mortality	2 (9.1)	11 (14.7)	0.726	13 (13.4)

^*^Values and percentages refer to the patient numbers in parentheses

ACPA: Anti-cytrulline peptide antibody, ANA: Anti-nuclear antibody, CRP: C-reactive protein, DLCO: Diffusing capacity of the lungs for carbon monoxide, ESR: Erythrocyte sedimentation rate, FVC: Forced vital capacity, ILD: Interstital lung disease, IP: Interstitial pneumonia, IQR: Interquartile range, LIP: lymphocytic interstitial pneumonia, MPO ANCA: Myeloperoxidase-specific antineutrophil cytoplasmic antibody, NSIP: Nonspesific interstitial pneumonia, OP: organizing pneumonia, PR3-ANCA: Serum anti-proteinase 3 antineutrophil cytoplasmic antibody, RD-ILD: Rheumatic disease-related interstital lung disease, RF: Rheumatoid factor, SD: Standart deviation, UIP: Usual interstitial pneumonia

Pulmonary symptoms were present in more than half of the patients in both groups. In the RD-ILD group, cough and dyspnea were observed with equal frequency, whereas cough was more prevalent in the non-RD-ILD group (Table 2). Raynaud's phenomenon and xerostomia were more frequent in RD-ILD patients (p = 0.010, p = 0.008), whereas xerophthalmia was comparable between the two groups (p = 0.068).

The ANA, RF, and ACPA positivity was higher in RD-ILD patients (p = 0.029, p = 0.003, p = 0.001, respectively). The ESR and CRP levels were similar between the two groups.

Indeterminate UIP and probable UIP (pUIP) patterns were included in the UIP group for comparison. The UIP pattern was more frequent in non-RD-ILD patients (77% vs. 54.5%); however, this difference was not statistically significant. In addition, there was no difference between NSIP pattern of RD-ILD patients and non-RD-ILD patients (22.7%, 9.3%, respectively).

In 11 patients, chest CT/HRCT showed a pattern consistent with probable UIP or fibrotic NSIP. These findings are presented separately in Table 2. Organizing pneumonia was not observed in any of the cases.

Diffusing capacity of the lungs for carbon monoxide (DLCO, %) results were available for 45 patients, while data were missing for 52 patients. The mean DLCO value was 61.2% (SD: 18.9, n = 14) in RD-ILD patients and 67.6% (SD: 19.7, n = 31) in non-RD-ILD patients. Forced vital capacity (FVC, %) results were assessed in 52 patients, whereas data were unavailable for 45 patients. The median FVC was 74.5% (range: 45–115) in RD-ILD patients and 81% (range: 39–74) in non-RD-ILD patients.

Among a total of 99 patients, mortality was observed in 13 (13.1%) cases. Two deaths occurred in the RD-ILD group while 11 were in the non-RD-ILD group. The exact causes of mortality could not be determined in the hospital medical records; however, 2 patients had malignancies (bladder and lung cancer).

#### Patients diagnosed with rheumatic disease-related ILD (RD-ILD)

A total of 22 (22.2%) patients were diagnosed with a rheumatic disease. The demographic, clinical, laboratory, imaging, and treatment findings of these RD-ILD patients are shown in Table 3.

**Table 3:** Clinical and laboratory findings of patients with rheumatic disease-related interstitial lung disease

Patients	Diagnosis	Sex	Age at ILD (y)	Smoking history	Chest CT/HRCT patterns	ANA	ENA	RF/ACPA^*^	ANCA	Treatment
1	pSS	F	58	Former smoker (10 p/y)	NSIP	Cytoplasmic	Anti-SSA	RF: low titer +	Negative	LD-GC + MMF
2	pSS	M	70	Former smoker (40 p/y)	UIP	Negative	Negative	RF: low titer +	Negative	MMF
3	pSS	F	58	Current smoker (15 p/y)	NSIP	Speckled	Anti-SSA52	Negative	Negative	-
4	pSS	F	71	Never	UIP	Speckled	Anti-SSA	RF: high titer +	Negative	MMF
5	pSS	F	75	Unknown	UIP	Negative	Negative	RF: high titer +	MPO-ANCA	AZA, LD-GC + MMF
6	pSS	F	75	Current smoker	UIP	Negative	Negative	Negative	Negative	AZA
7	pSS	F	74	Never	LIP	Speckled	Anti-SSA Anti-SSB	Negative	Negative	MMF
8	Limited cutaneous SSc	F	54	Never	UIP	Centromer	Negative	Negative	Negative	AZA, MMF
9	Diffuse cutaneous SSc	F	57	Never	pUIP	Speckled	Negative	Negative	Negative	LD-GC, CYC, MMF
10	Limited cutaneous SSc	F	67	Never	pUIP or fibrotic NSIP	Nucleolar	Anti-Scl70	Negative	Negative	CYC
11	Limited cutaneous SSc	F	48	Never	UIP	Centromer	Anti-Scl70	Unknown	Unknown	MD-GC + CYC, MMF
12	Limited cutaneous SSc	F	61	Never	pUIP or fibrotic NSIP	Homogenous	Anti-Scl70	Negative	Negative	MMF
13	RA	M	44	Current smoker	pUIP or fibrotic NSIP	Negative	Negative	Negative	Negative	LD-GC, CYC, MMF, RTX, nintedanib
14	RA	M	67	Former smoker (35p/y)	UIP	Negative	Negative	ACPA: high titer +	Negative	Unknown
15	RA	F	62	Current smoker	NSIP	Negative	Negative	RF: high titre +	Negative	LD-GC, HQ, MTX, TCZ
16	RA	M	56	Current smoker	NSIP	Negative	Negative	ACPA: high titer +	Negative	LD-GC
17	RA	M	65	Unknown	UIP	Negative	Negative	ACPA: high titer +	Negative	RTX
18	SLE	F	73	Unknown	UIP	Cytoplasmic	Anti-SSA Anti-SSA52	Unknown	Negative	HD-GC, CYC, RTX, MMF
19	SLE	F	75	Never	NSIP	Positive	Anti-Sm	Unknown	Unknown	HQ
20	UCTD	F	70	Never	pUIP	Positive	Anti-Sm	ACPA: low titer +	Negative	LD-GC + AZA
21	UCTD	F	78	Never	UIP	Cytoplasmic	Anti-Scl70	Unknown	Negative	-
22	MPA	M	63	Never	pUIP or fibrotic NSIP	Negative	Negative	ACPA: high titer +	MPO-ANCA	IV pulse GC, HD-GC, RTX

ACPA: Anti-cytrulline peptide antibody, ANA: Anti-nuclear antibody, ANCA: anti-neutrophil cytoplasmic antibody, AZA: Azathioprine, CYC: Cyclophosphomide, ENA: Extractable nuclear antigen, GC: glucocorticoid, HD-GC: High-dose glucocorticoid, HQ: hydroxychloroquine, LD-GC: low-dose glucocorticoid, LIP: lymphocytic interstitial pneumonia, MD-GC: medium-dose glucocorticoid, MMF: mycophenolate mofetil, MPA: Microscopic polyangiitis, NSIP: Nonspesific interstitial pneumonia, pSS: primary Sjögren’s syndrome, pUIP: probable UIP, RA: Rheumatoid arthritis, RF: Rheumatoid factor, RTX: Rituximab, SLE: Systemic lupus erythematosus, SSc: Systemic sclerosis, UCTD: Undifferantiated connective tissue disease, UIP: Usual interstitial pneumonia, TCZ: Tocilizumab

^*^RF and ACPA levels ≥ 3 times the upper limit of normal (ULN) were considered high titer, while levels < 3 × ULN were considered low titer

##### Primary Sjögren’s syndrome

Seven (7.1%) patients were diagnosed with pSS. All patients exhibited sicca symptoms. Three tested positive for anti-SSA and/or anti-SSB, and one was positive for Anti-Ro52 antibodies, while the other 3 had diagnostic findings on minor salivary gland biopsy. Six patients had a Schirmer test result of ≤ 5 mm in at least one eye. Although the minor salivary gland biopsy result for one patient was uavailable, the diagnosis was made based on the evaluating clinician's expert opinion with current clinical signs and symptoms.

The median age of diagnosis was 71 years (IQR: 17), and all were female. Chest CT/HRCT scans revealed a UIP pattern in 4 cases, an NSIP pattern in 2 cases, and an LIP pattern in only 1 case. Among the 2 patients with NSIP patterns on chest CT/HRCT, 1 received low-dose glucocorticoids in combination with mycophenolate mofetil (MMF), and the other did not receive any treatment for ILD. The patient with an LIP pattern on CT was treated with MMF alone. Three patients with UIP patterns on chest CT/HRCT received MMF, 1 in combination with low dose glucocorticoids, and one patient received azathioprine (AZA).

##### Rheumatoid arthritis

Among the 5 patients diagnosed with RA, 4 (80%) were seropositive, all had joint involvement concomitantly, and 3 (60%) exhibited elevated ESR and CRP levels. The mean age of diagnosis was 58.8 years (SD: 9.2), and 4 (80%) patients were male. All patients with RA had a history of tobacco use.

In terms of chest CT/HRCT patterns, 2 patients had NSIP, 2 had UIP, and 1 had probable UIP or fibrotic NSIP. Patients with an NSIP pattern on chest CT/HRCT (n = 2) received glucocorticoids, and one of them additionally received tocilizumab. The patient with probable UIP or fibrotic NSIP on chest CT/HRCT was treated with cyclophosphamide, MMF, and rituximab in combination with glucocorticoids. Data for 2 patients with UIP patterns on chest CT/HRCT were unavailable.

##### Systemic sclerosis

Among the 5 patients diagnosed with SSc, 1 had diffuse cutaneous SSc, while 4 had limited cutaneous SSc. The mean age of diagnosis was 57.4 years (SD: 7.1), and all were female. None of the patients had a history of tobacco use.

Chest CT/HRCT scans showed a UIP pattern in 3 patients and, a probable UIP or fibrotic NSIP pattern in 2 patients with SSc. Anti-Scl-70 antibodies were positive in two patients; one patient tested positive for anti-centromere antibodies, and one tested positive for both. Despite the absence of positive autoantibody results, one patient exhibited skin thickening of the fingers of both hands extending proximal to the metacarpophalangeal joints, with additional involvement of the trunk. Of 3 patients, 2 received moderate-dose glucocorticoids, while one received a mild dose. One patient was treated with cyclophosphamide, 2 received cyclophosphamide and MMF, one was treated with MMF alone, and one received AZA and MMF at different times.

##### Systemic lupus erythematosus

Two patients (2%) were diagnosed with SLE, and both were female. One patient demonstrated a UIP pattern on chest CT/HRCT and received high-dose glucocorticoids, cyclophosphamide, MMF, and rituximab. The other had an NSIP pattern and did not use immunosuppressive therapy.

##### Undifferentiated connective tissue disease (UCTD)

Both patients were female and exhibited nonspecific autoimmune features, including arthralgia and sicca symptoms, and tested positive for ANA. One patient was also positive for anti-Sm/RNP and ACPA antibodies. The other patient had anti-Scl-70 antibody positivity but did not exhibit any clinical features consistent with systemic sclerosis. One patient had a pUIP pattern on chest CT/HRCT and was treated with low-dose glucocorticoids and AZA. The other patient demonstrated a UIP pattern and was managed without immunosuppressive therapy. No progression to a defined CTD was observed during follow-up.

##### Vasculitis

Only 1 (1%) patient was diagnosed with MPA. This patient had ILD, acute renal failure, proteinuria, and a high titer of MPO-ANCA positivity. The chest CT/HRCT patterns indicated probable UIP or fibrotic NSIP. He was treated with pulse methylprednisolone and rituximab.

##### IPAF

Among the 99 ILD patients, 2 met the IPAF classification criteria [8]. ANA testing was positive in both of the cases; 1 patient exhibited a nucleolar pattern with a titer of 1:100, while the other showed a cytoplasmic pattern with a titer of 1:1000–1:3200. Chest CT/HRCT imaging demonstrated an NSIP pattern in both patients. The female patient presented with Raynaud's phenomenon and cough. Nailfold capillaroscopy findings were nonspecific, and her DLCO level was moderately reduced (72%). The male patient also had a cough, with a significantly decreased FVC (54%). Both patients were current smokers and had not received any immunosuppressive therapy.

## Discussion

In this study, we aimed to assess the presence of rheumatologic disease in patients referred to the rheumatology clinic with suspected ILD. Among the patients with suspected ILD, those with IIPs patterns were selected. Notably, approximately one-third of the patients (30.8%) ILD diagnosis could not be confirmed, highlighting the importance of careful imaging review, experienced radiologist in pulmonology and differential diagnosis. Among non-ILD patients, 7.7% were considered to have hypersensitivity pneumonitis, while others exhibited a wide range of imaging findings, including infection to congestion.

Our findings revealed that 22.2% of ILD patients were diagnosed with a systemic rheumatic disease, with pSS being the most common. The UIP pattern was predominant, particularly among non-RD-ILD patients. Autoantibody positivity was also significantly higher in RD-ILD patients. In the literature, the prevalence of RD-ILD is approximately 30% [6, 7]; however, in our results, this rate was found to be lower. This discrepancy may be attributed to increased awareness of RD-ILD over the years and improved accessibility to rheumatologists which results in an earlier diagnosis of rheumatologic disease before ILD diagnosis. The other explanation for the lower prevalence is that a single cross-sectional evaluation may be insufficient for diagnosis.

Several previous studies have reported varying prevalence rates of ILD in systemic rheumatic diseases. The prevalence has been estimated at 34.7% in limited SSc, and 53.4% in diffuse SSc [17], various estimates ranging between 10 to 60% in pSS [18], between 10 to 60% in RA [19], between 52 to 67% in mixed connective tissue disease [20]. A study found out that 2.7% of patients with systemic vasculitis (primarily MPA) had ILD, and 2 of these patients were diagnosed with ILD before the diagnosis of vasculitis [21]. In our study pSS was the most common etiology for RD-ILD. It frequently presents with occult or subtle clinical symptoms and may result in underdiagnosis until pulmonary involvement. Only 1 patient (1%) was diagnosed with MPA in our cohort who presented with ILD.

Clinical signs of autoimmune disease should prompt early rheumatologic evaluation. In our study, Raynaud’s phenomenon and xerostomia were significantly more prevalent in RD-ILD patients, suggesting that even mild autoimmune symptoms may indicate an underlying CTD. Given that ILD may precede systemic rheumatic disease onset, it is crucial to recognize early and isolated pulmonary involvement. Our findings emphasize that rheumatologic diseases should always be considered in ILD patients as an initial sign of CTD, even in the absence of overt systemic manifestations. A study observed that patients diagnosed with IIPs developed CTD symptoms over a period of 11 years [22]. In addition, two different studies reported that 15% [7] and 25% [23] of new CTD diagnoses were made among ILD patients. Kono et al. reported that patients with prior diagnosis of idiopathic NSIP developed CTD after 2 years of ILD diagnosis [24]. Recent cohorts from Denmark, the United States, and China reported that 10–17% of RA-ILD patients were diagnosed with ILD before the diagnosis of RA, and 7–34% were diagnosed concurrently with RA, suggesting that ILD can precede RA onset [25]. Our findings align with previous reports, 22.2% of ILD patients were diagnosed with a rheumatic disease after evaluation. These findings highlight the importance of early rheumatologic screening in patients with suspected ILD, including symptom review, serologic testing, and imaging pattern recognition, even in the absence of autoimmune symptoms. A multidisciplinary approach and regular follow-up in indeterminate cases may facilitate earlier diagnosis of rheumatic diseases and improve patient outcomes.

It is well established in the literature that NSIP is the predominant HRCT pattern in CTD whereas the UIP pattern is more frequently observed in RA [2, 26]. However, in our cohort, the UIP pattern was the predominant CT finding, observed in 77% of non-RD-ILD patients and 54.5% of RD-ILD patients. This discrepancy may be due to selection bias, as our study primarily included patients who were referred from a pulmonologist for suspected ILD rather than those with established rheumatic disease.

Another key observation in our study was the strong association between smoking and fibrotic ILD patterns. The UIP pattern, commonly linked to more progressive and irreversible lung disease, was significantly more frequent in non-RD-ILD patients, who also had a higher prevalence of tobacco use (p = 0.006). This supports prior evidence that smoking plays a role in driving a more fibrotic disease course [27].

Furthermore, our study reinforces the importance of serological markers in ILD evaluation. ANA, RF, and ACPA positivity were significantly higher in RD-ILD patients. These findings are consistent with previous literature, indicating that RF and ACPA positive RA patients, as well as Anti-Scl-70-positive SSc patients, have higher risk of ILD [28–30]. Additionally, studies had reported that ANA and Anti-Ro52 antibody positivity was associated with ILD in patients with pSS and mixed connective tissue disease [18, 31–33]. These findings suggest that autoantibody testing should be a part of ILD workup, particularly in patients with suggestive clinical features.

In our study, 31.3% of patients with a confirmed ILD diagnosis were asymptomatic at the time of diagnosis, emphasizing the need for increased awareness regarding subclinical ILD. Asymptomatic ILD is often detected incidentally through imaging performed for other medical reasons, such as routine health screenings or evaluations for unrelated conditions. The presence of asymptomatic ILD is particularly relevant in rheumatic diseases, where since ILD can precede systemic manifestations. Asymptomatic patients may still exhibit early structural lung changes [34, 35], including subclinical fibrosis or mild reductions in pulmonary function tests, which could eventually progress to symptomatic disease [36]. Therefore, monitoring patients with CTD for ILD was suggested [37].

Interestingly, only 2 patients in our study met the IPAF classification criteria [8], which may be attributed to the predominance of UIP findings in the non-rheumatic disease group and the small sample size. Another possible reason for the low number of patients could be missing serological data. In addition, the IPAF classification criteria [8] were established in 2015; prior to this, patients may have been evaluated based on symptoms and physical examination findings rather than from an IPAF perspective, and autoantibodies may have been tested accordingly for differential diagnosis. Both patients were excluded from the comparative analysis to maintain diagnostic clarity between well-defined RD-ILD and non-RD-ILD groups. While the IPAF group represents an important clinical entity with features suggestive of CTD, it is not considered a definitive CTD diagnosis in the current literature and the small number of patients (n = 2) limited the feasibility of meaningful subgroup comparison. These patients were described separately to highlight their potential significance within the broader spectrum of autoimmune-related ILD.

Our study has several limitations. First, its retrospective design led to missing data. Other limitations of this study are the small size of population, and the lack of treatment follow-up data for non-RD-ILD patients. As some of these patients were lost to follow up, their therapeutic approaches and long-term outcomes remain unknown. We do not know whether findings would be detected in repeated tests in these patients or whether they would develop rheumatic disease during follow-up. Finally, although chest CT/HRCT scans were reviewed, not all imaging was high-resolution CT, which may have influenced the findings. In addition, all chest CT/HRCT images were evaluated by a single thoracic radiologist. Although the radiologist is highly experienced in thoracic imaging and interstitial lung disease patterns, the lack of a second independent reviewer introduces the possibility of interpretive subjectivity. Future studies would benefit from involving multiple blinded radiologists to enhance diagnostic reliability and evaluate inter-observer variability.

## Conclusion

Our study highlights that a significant proportion of ILD patients were later diagnosed with a systemic rheumatic disease. This underscores the importance of evaluating ILD patients for underlying autoimmune conditions, even in the absence of overt rheumatic symptoms. The patients diagnosed with RD-ILD in our study had no prior rheumatic disease diagnosis at the time of their ILD diagnosis. This suggests that ILD can be an initial or even the sole manifestation of an underlying systemic autoimmune disease. Approximately one-third of the patients admitted to the rheumatology outpatient clinic with a diagnosis of ILD were not verified, implying the need for radiologists experienced in pulmonary diseases.

These findings emphasize the need for routine autoimmune screening in ILD patients, particularly in those with unexplained lung involvement.

## Data Availability

All data generated or analyzed are available from the corresponding author on reasonable request.
